# Supplementary material for: VEGFA activates an epigenetic pathway upregulating ovarian cancer‐initiating cells
Source: EMBO Mol Med. 2017 Feb 8;9(3):304–18. doi: 10.15252/emmm.201606840 (PMC5331266; doi:10.15252/emmm.201606840)
Supplement: Supplementary file 1 — Expanded View Figures PDF [file EMMM-9-304-s001.pdf]

Expanded View Figures

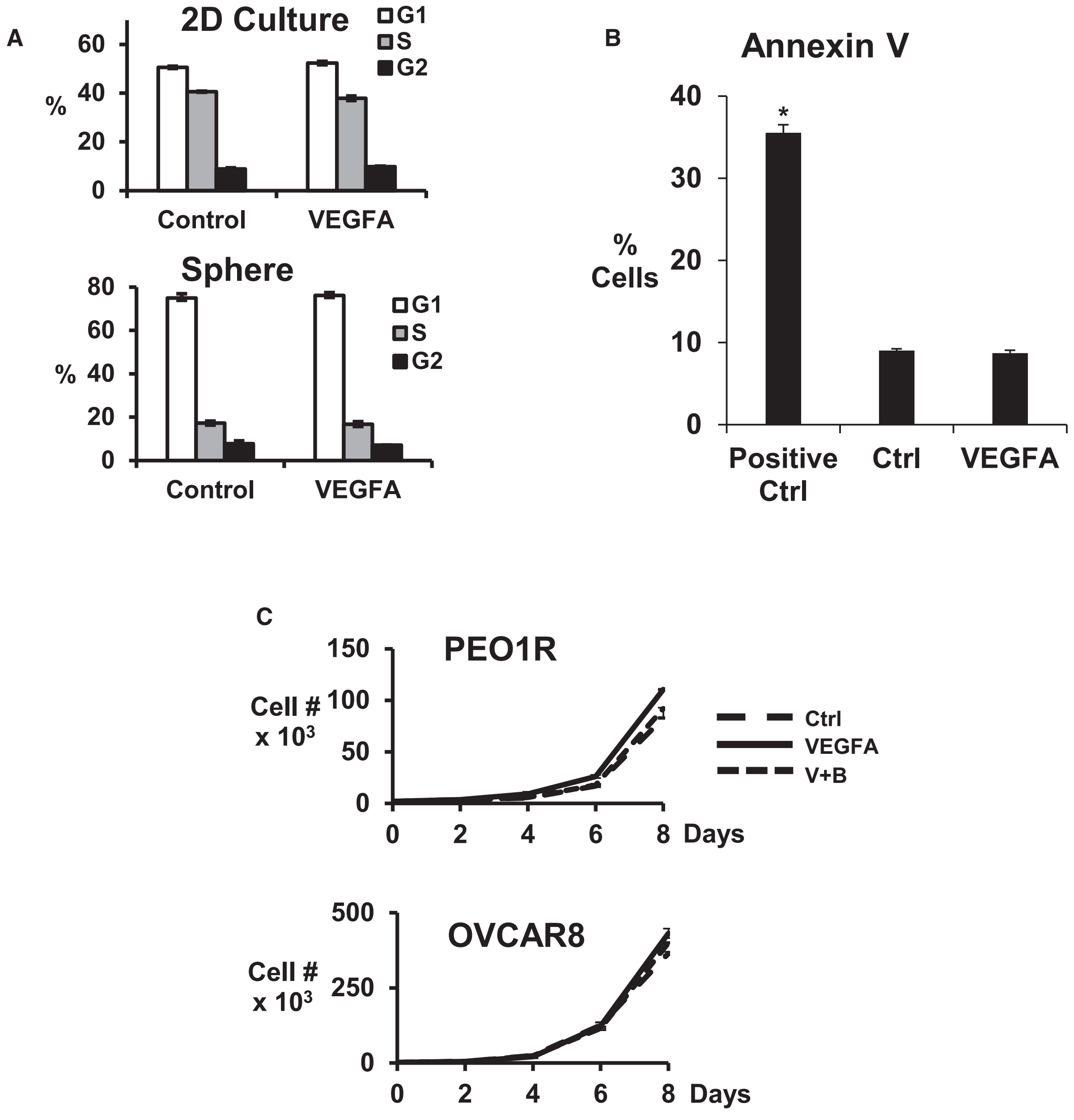

Figure EV1. VEGFA does not change cell cycle, apoptosis or cell proliferation.

A The cell cycle distributions of OVCAR8 cells treated without (control) or with VEGFA in 2-day culture over 7 days (top) or assayed from dissociated OVCAR8 spheres formed ± VEGFA (bottom panel). Similar findings were observed with PEO1R cells (not shown).

B PEO1R cells were treated with or without VEGFA for 7 days and then recovered for annexin V-FITC/PI flow cytometric analysis to quantitate the % of apoptotic cells. Positive control cells were treated with 200 nM paclitaxel. *P = 0.0001.

C Equal numbers (10,000 cells) of PEO1R and OVCAR8 cells were plated into 2-day culture ± VEGFA, and viable cells were counted every 2 days. Triplicate repeat data show no difference in population growth between groups over 8 days.

Data information: All graphed data show mean ± SEM for at least 3 different biologic experiments with at least three technical repeats within each assay. Differences between multiple treatment groups were compared by ANOVA.

 © 2017 The Authors

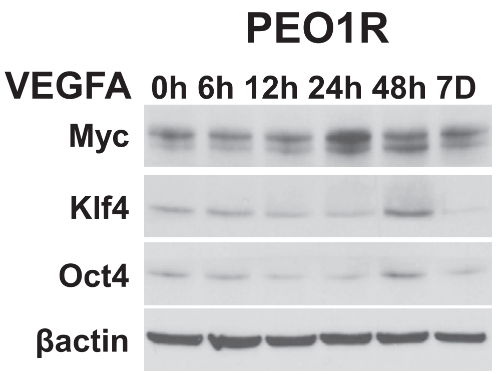

Figure EV2. VEGFA effects on embryonic stem cell transcription factors.
Western blot of VEGFA effects on indicated proteins in PEO1R cells at indicated times in hours (h) or days (D).

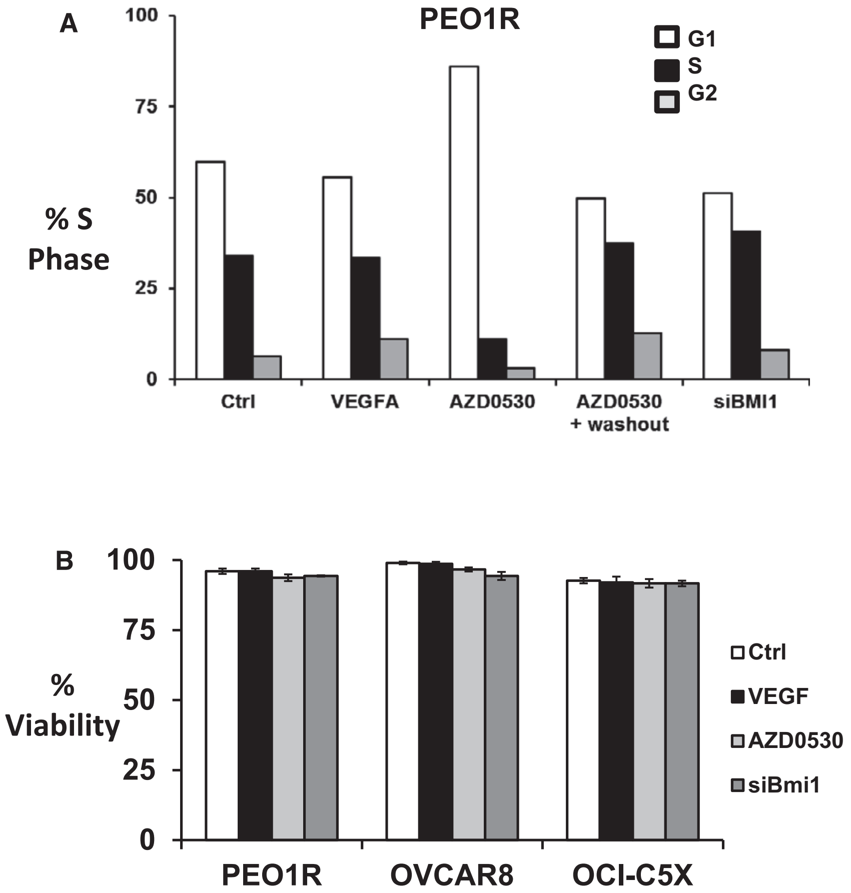

Figure EV3. Cell cycle distribution and viability of cells used in sphere assays and/or in tumor-initiating stem cell assays.

A Cell cycle distribution was assayed immediately prior to plating into sphere formation or prior to injection into nude mice for limiting dilution stem cell assays. Cells were recovered for cell cycle distribution after either 7 days of VEGFA followed by 2 days without cytokine (VEGFA), or after 7-day treatment with VEGFA with AZD0530 added for 48 h (days 6 and 7) prior to a 2-day washout without cytokine or AZD0530 (AZD0530 + washout). siBMI1 cells were transfected with siBMI1 for 48 h prior to addition of VEGFA for 7 days and followed by 2 days without cytokine. While AZD0530 (1 μM) over 48 h caused partial G1 arrest (AZD0530), cells return to asynchronous cycling after a 2-day washout without AZD0530 (AZD0530 + washout).

B PEO1R cell viability was not changed by 1 week of VEGFA exposure with or without either Src inhibition in the last 48 h of treatment, or by prior knockdown of Bmi1 48 h prior to addition of VEGFA. All graphed data show mean ± SEM for at least 3 different biologic experiments with at least three technical repeats within each assay.

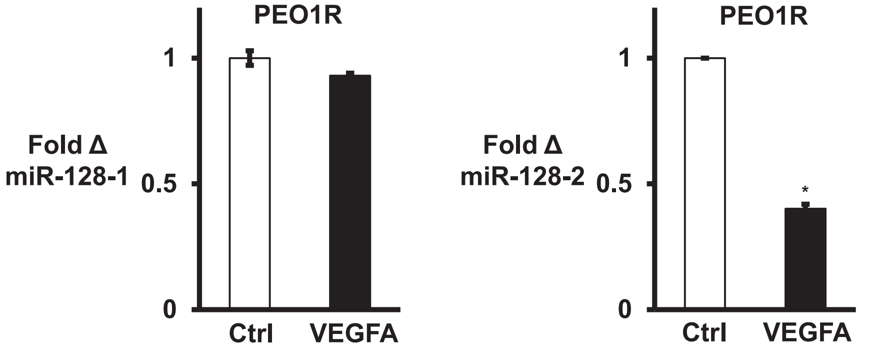

Figure EV4. VEGFA decreases *miR-128-2* expression but not that of *miR-128-1*.

PEO1R cells were treated with VEGFA for 7 days and then *miR-128-1* and *miR-128-2* levels assayed by qPCR. Data are graphed as mean ± SEM. All assays were performed in triplicate biologic experiments with at least three technical repeats within each assay. Differences between two groups were assayed by Student's *t*-test. *P = 0.00011.

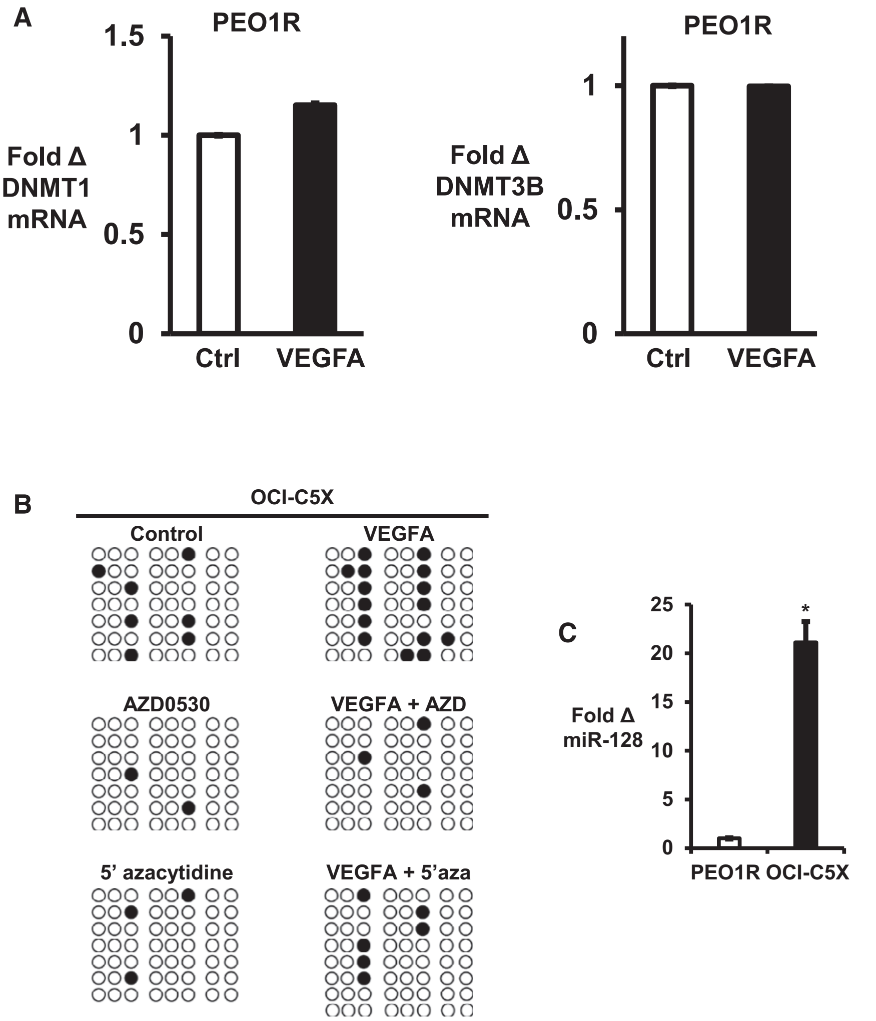

Figure EV5. VGFA effects on DNMT levels and methylation of *miR-128-2*.

- A DNMT1 and DNMT3B expression levels are not affected by VEGFA treatment over 7 days in PEO1R.
- B Results of bisulfite sequencing of the *miR-128-2* region in OCI-C5X. Open circles indicate unmethylated, and filled circles indicated methylated CpG sites.
- C Expression levels of miR-128 in PEO1R and OCI-C5X are graphed. *P = 0.0007. Differences between two groups were assayed by Student's *t*-test.

Data information: All graphed data show mean ± SEM for at least 3 different biologic experiments with at least three technical repeats within each assay.

 © 2017 The Authors

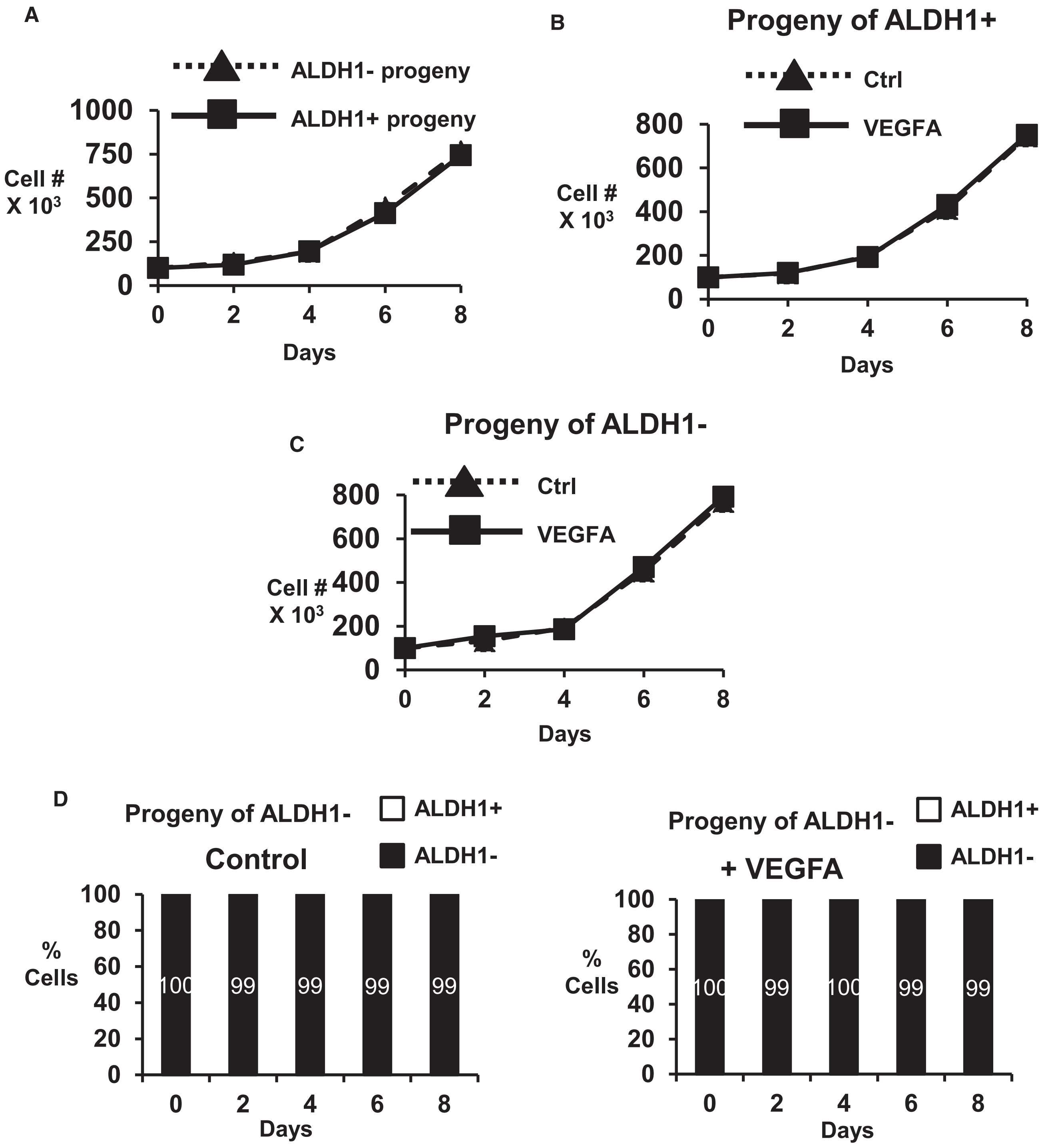

Figure EV6. Population growth of ALDH1$^+$ and ALDH1$^-$ cells without and with VEGFA.

A Population growth from sorted ALDH1$^-$ and ALDH1$^+$ PEO1R cells.
B Population growth from sorted ALDH1$^+$ cells with and without VEGFA.
C Population growth from sorted ALDH1$^-$ cells with and without VEGFA.
D Proportions of ALDH1$^-$ or ALDH1$^+$ cells arising from ALDH1$^-$ cells. ALDH1$^-$ cells only give rise to ALDH1$^-$ progeny and do not yield ALDH1$^+$ cells.

© 2017 The Authors
